# Supplementary material for: Molecular Screening for High-Risk Human Papillomaviruses in Patients with Periodontitis
Source: Viruses. 2023 Mar 22;15(3):809. doi: 10.3390/v15030809 (PMC10059129; doi:10.3390/v15030809)
Supplement: Supplementary file 1 [file viruses-15-00809-s001.zip › viruses-2301653-supplementary.pdf]

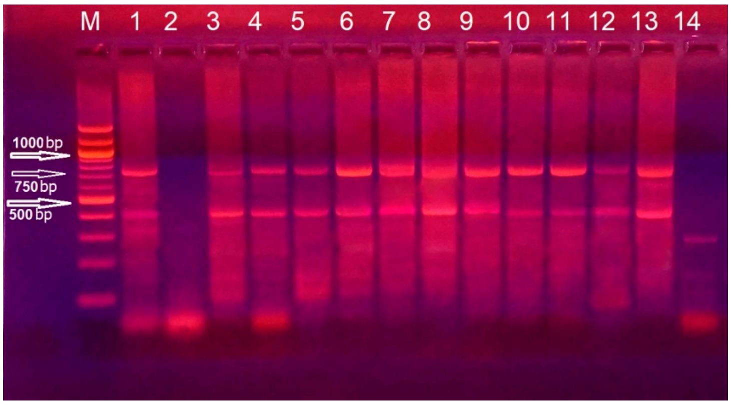

Figure S1. Gel electrophoresis of the HPV positive samples (lines 1, 3-13) with primers GPE6/5B/6B (700 bp) and MY09/11 (450 bp). M – 100 bp DNA ladder; Line 2 – negative control; 14 – beta globin (268 bp).
